# Plasma TIMP-1 as a sex-specific biomarker for acute lung injury

**DOI:** 10.1186/s13293-022-00481-9

**Published:** 2022-12-08

**Authors:** Sultan Almuntashiri, Timothy W. Jones, Xiaoyun Wang, Andrea Sikora, Duo Zhang

**Affiliations:** 1 Clinical and Experimental Therapeutics, College of Pharmacy, University of Georgia and Charlie Norwood VA Medical Center, Augusta, GA 30912 USA; 2 Department of Clinical Pharmacy, College of Pharmacy, University of Hail, Hail, 55473 Saudi Arabia; 3 Department of Clinical and Administrative Pharmacy, College of Pharmacy, University of Georgia, Augusta, GA 30901 USA; 4 Department of Pharmacy, Augusta University Medical Center, Augusta, GA 30912 USA; 5 Department of Medicine, Augusta University, Augusta, GA 30912 USA

**Keywords:** Acute respiratory distress syndrome, Mortality, Matrix metalloproteinases, Critical illness

## Abstract

**Background:**

Acute respiratory distress syndrome (ARDS) confers high morbidity and mortality, with a death rate reaching 40%. Pre-clinical and clinical studies have cited sex-specific sex hormones as a critical contributor to divergent immunologic responses. Therefore, exploration of sex and sex hormone roles following lung injury and ARDS development is needed. Tissue inhibitor of metalloproteinase-1 (TIMP-1) was the first-discovered natural collagenase inhibitor and is located exclusively on the X chromosome. This study aimed to evaluate the prognostic role of circulating TIMP-1, and if concentration differences between males and females correlate with the mortality of ARDS patients.

**Methods:**

Human plasma samples from 100 ARDS patients enrolled in Albuterol to Treat Acute Lung Injury (ALTA) trial on the day of randomization were evaluated. The amount of TIMP-1 was measured using an enzyme-linked immunoassay (ELISA). Area under the receiver operating characteristic (AUROC) was computed to assess the predictive power of TIMP-1 for 30 and 90-day mortality. Chi-squared tests and Kaplan–Meier curves were computed to assess different variables and survival.

**Results:**

AUROC analysis of TIMP-1 and 30-day mortality among females showed that TIMP-1 exhibited an AUC of 0.87 (95% confidence interval [CI] 0.78 to 0.97; *P* = 0.0014) with an optimal cut-off value of 159.7 ng/mL producing a 100% sensitivity and 74% specificity. For 90-day mortality, AUROC analysis showed an AUC of 0.82 (95% confidence interval [CI] 0.67 to 0.97; *P* = 0.0016) with a similar cut-off value producing a 90% sensitivity and 76.47% specificity. Stratifying subjects by TIMP-1 concentration as high (≥ 159.7 ng/mL) or low (< 159.7 ng/mL) indicated that high TIMP-1 was associated with increased 30 and 90-day mortality rates (all *P* < 0.0001). Lastly, high TIMP-1 group was associated with worse other outcomes including ventilator-free days (VFDs) and ICU-free days (all *P* < 0.05).

**Conclusion:**

Circulating TIMP-1 appeared to be a promising biomarker for mortality among females with ARDS. The high TIMP-1 group showed worse VFDs and ICU-free days. Circulating TIMP-1 may be a sex-specific biomarker in the setting of ARDS and could improve ARDS phenotyping as well as provide a novel therapeutic target in females.

## Highlights

- Plasma TIMP-1 levels are significantly elevated in ARDS patients.
- Plasma TIMP-1 levels show no difference between female and male ARDS patients.
- Considering the 30 and 90-day mortality, female non-survivors have significantly higher plasma TIMP-1 levels compared to female survivors. However, there is no difference between non-survivors and survivors in the male group.
- Plasma TIMP-1 levels demonstrate an excellent discriminating ability for the prediction of mortality among female ARDS patients.
- The high TIMP-1 level group shows worse other relevant clinical outcomes, VFDs, and ICU-free days.

## Introduction

Acute lung injury (ALI) and acute respiratory distress syndrome (ARDS) are life-threatening diseases affecting 200,000 patients yearly in the United States and roughly 10% of all patients during intensive care unit admissions [1–5]. Despite modern advances in diagnosis and therapy for ARDS over the last 50 years, mortality has remained high at 30% to 40% [6]. The lack of diagnostic or prognostic biomarkers has stalled detection methods and effective treatment development [7]. ARDS-specific biomarkers will aid in defining ARDS, and determining patient phenotypes most likely experience a therapeutic benefit [8].

The inflammatory response to lung injury occurs through multiple mechanisms with demographic, clinical, and genetic characteristics contributing. Sex has been assessed in relation to ARDS morbidity and mortality with inconsistencies in the literature. In a large prospective cohort of critically injured adults, women were more likely than men to develop ARDS, but the mortality did not differ according to gender among ARDS patients [9]. Moreover, a retrospective cohort of acute respiratory failure patients has shown that the female sex was associated with more mortality in patients with severe ARDS [10]. These studies suggested that sex hormones could directly or indirectly contribute to the development of ARDS as well as both morbidity and mortality. On the other side, the male gender appears to be a prominent risk for ARDS development and mortality in a large retrospective cohort of trauma patients who developed ARDS [11]. Similarly, the average rate of ARDS-caused mortality was higher in males than females in a large cohort with a long follow-up period over 15 years [12]. One study showed no significant difference between male and female patients with acute respiratory failure in all-cause mortality as well as other clinical outcomes, including duration of mechanical ventilation, ICU length of stay, and hospital length of stay [13]. These studies also emphasized the heterogeny regarding sex-based morbidity and mortality in ARDS.

The inflammatory response to lung injury propagates from multiple factors with pathologic synergy leading to the varying severity and phenotypes of ARDS [9]. Pre-clinical and clinical studies suggest that sex hormones account for differing immunologic responses [14–16]. In particular, pro-inflammatory estrogens, and immune function depressing testosterone were identified as potential explanations for the severity of ARDS in female patients [9]. Furthermore, the female sex has been recognized as a risk factor for hospitalization in COPD patients [17] and the development of asthma [18]. Collectively, these studies suggested that females appeared to be more prone to the development of pulmonary diseases. Sex-specific biomarkers from this perspective could be valuable in future research on ARDS and encourage further exploration of the role of sex following lung injury.

TIMPs (tissue inhibitor of metalloproteinases) control the enzymatic activity of matrix metalloproteinases (MMPs) and are well-known for regulating extracellular matrix (ECM) turnover [19]. TIMP-1 was the first-discovered natural collagenase inhibitor and has a genomic location exclusive to the X chromosome [20, 21]. Different from many X-linked genes, Anderson and colleagues reported that human TIMP-1 is prone to reactivation or variable in its inactivation [22]. In addition, it has been reported that estradiol can significantly induce TIMP-1 expression in goat oviductal epithelial cells [23] and human aortic endothelial cells under inflammatory conditions [24]. Thus, there is a possibility that TIMP-1 may serve as a sex-specific biomarker. TIMP-1 is ubiquitously expressed in numerous human cells and tissues [25]. Studies have shown that TIMP-1 from fibroblasts and immune cells contributes to the pathogenesis of lung diseases [26, 27].

Recently, a novel investigation of TIMP-1 found elevated systemic TIMP-1 was associated with worse hypoxemia and increased 90-day mortality in a large cohort of mechanically ventilated ARDS patients with respiratory failure [28]. Nevertheless, TIMP-1 and its regulation and function in ALI/ARDS remain largely unknown. In the current study, we hypothesized that high levels of circulating TIMP-1 in female ARDS patients would be associated with more severe lung injury and worse outcomes.

## Materials and methods

### Study population

100 plasma samples from patients enrolled in Albuterol to Treat Acute Lung Injury (ALTA) trial on the day of randomization were obtained through the Biologic Specimen and Data Repository Information Coordinating Center (BioLINCC) of the National Heart, Lung and Blood Institute (NHLBI). Patients were grouped based on sex, with independent samples from 54 males and 46 females. 20 plasma samples from healthy donors were obtained from Innovative Research (Novi, MI) to serve as controls.

### Plasma measurement

Circulating TIMP-1 concentrations were measured using enzyme-linked immunoassay (ELISA) (R&D Systems, Minneapolis, MN) on the day of randomization.

### Statistical analysis

Statistical analyses were performed with IBM SPSS Statistics Version 27.0. Figures were developed using GraphPad Prism. The significance level was set at $\alpha =0.05.$ Descriptive statistics were used to describe demographic data. An area under the receiver operating characteristic (AUROC) analysis was computed to assess the predictive power of TIMP-1 for 30 and 90-day mortality, and determine the optimal cut-off plasma concentration of TIMP-1 using Youden’s index. Kaplan–Meier survival curves were computed for the TIMP-1 groups identified by the AUROC analysis, and a log-rank test was performed to evaluate survival differences between groups.

## Results

### Study population

The demographic and summary characteristics of ALTA sub-groups are shown in Table 1. A significant difference was found between male and female groups for age (*P* = 0.035). No significant differences were observed between male and female groups for the score of acute physiology and chronic health evaluation (APACHE III), PaO_2_/FiO_2_ ratio, and major outcomes of ALTA trials, including VFDs, ICU-free days, and organ failure-free days. For ARDS etiology, a significant difference was noticed between the groups with regard to trauma-induced lung injury (*P* = 0.016).

**Table 1:** Demographic and summary characteristics of ALTA sub-groups

Characteristic	Male (*n* = 54)	Female (*n* = 46)	*P* value
Age	47.74 ± 2.23	54.30 ± 2.042	0.035
APACHE III score	93.40 ± 3.83	90.96 ± 4.51	0.679
PaO_2_/FIO_2_	151.80 ± 8.76	130.44 ± 7.76	0.076
Primary cause of ARDS, %			
Aspiration	18.5	13	0.457
Multiple transfusion	1.9	2.2	0.909
Other	3.7	8.7	0.295
Pneumonia	38.9	37	0.843
Sepsis	20.4	37	0.066
Trauma	16.7	2.2	0.016
Ventilator-free days	13.91 ± 1.39	15.72 ± 1.50	0.378
ICU-free days	12.87 ± 1.18	14.63 ± 1.43	0.342
Organ failure-free days	14.59 ± 1.37	14.09 ± 1.65	0.813

Means and standard error of mean (SEM)s were reported for continuous variables

*APACHE III score* Acute Physiology, Age and Chronic Health Evaluation; *PaO*_*2*_ partial pressure of oxygen, *FIO*_*2*_ fraction of inspired oxygen

### Plasma TIMP-1 levels in normal subjects and ARDS patients

**Fig. 1:**
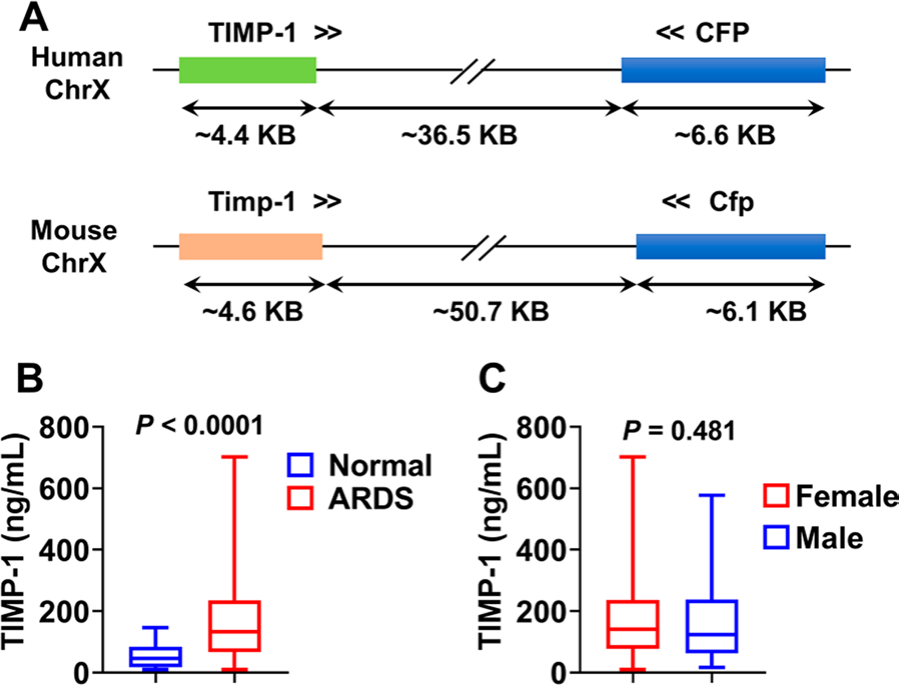
Plasma TIMP-1 levels in ARDS patients. **A** Genomic structures of mouse and human *TIMP-1*. **B** Plasma TIMP-1 levels from normal subjects (*n* = 20) and ARDS patients (*n* = 100). **C** Comparison of plasma TIMP-1 levels in female (*n* = 46) and males patients (*n* = 54)

X-chromosomal gene *TIMP-1* is highly conserved among mammals. The genomic structure of human *TIMP-1* and mouse *Timp-1* is shown in Fig. 1A. In our study, we measured plasma TIMP-1 from both ARDS patients and the normal control group. We found that plasma TIMP-1 levels were significantly higher in ARDS patients when compared with levels in normal subjects. The median level of TIMP-1 was 45.82 ng/mL in the control group (*n* = 20) and 132.5 ng/mL in ARDS group (*n* = 100), respectively (Fig. 1B, *P* < 0.0001 ARDS vs normal). Next, TIMP-1 levels were compared between female and male ARDS patients, but no significant difference is observed (Fig. 1C, *P* = 0.481 females vs males).

### Plasma TIMP-1 level and association with 30- and 90-day mortality

**Fig. 2:**
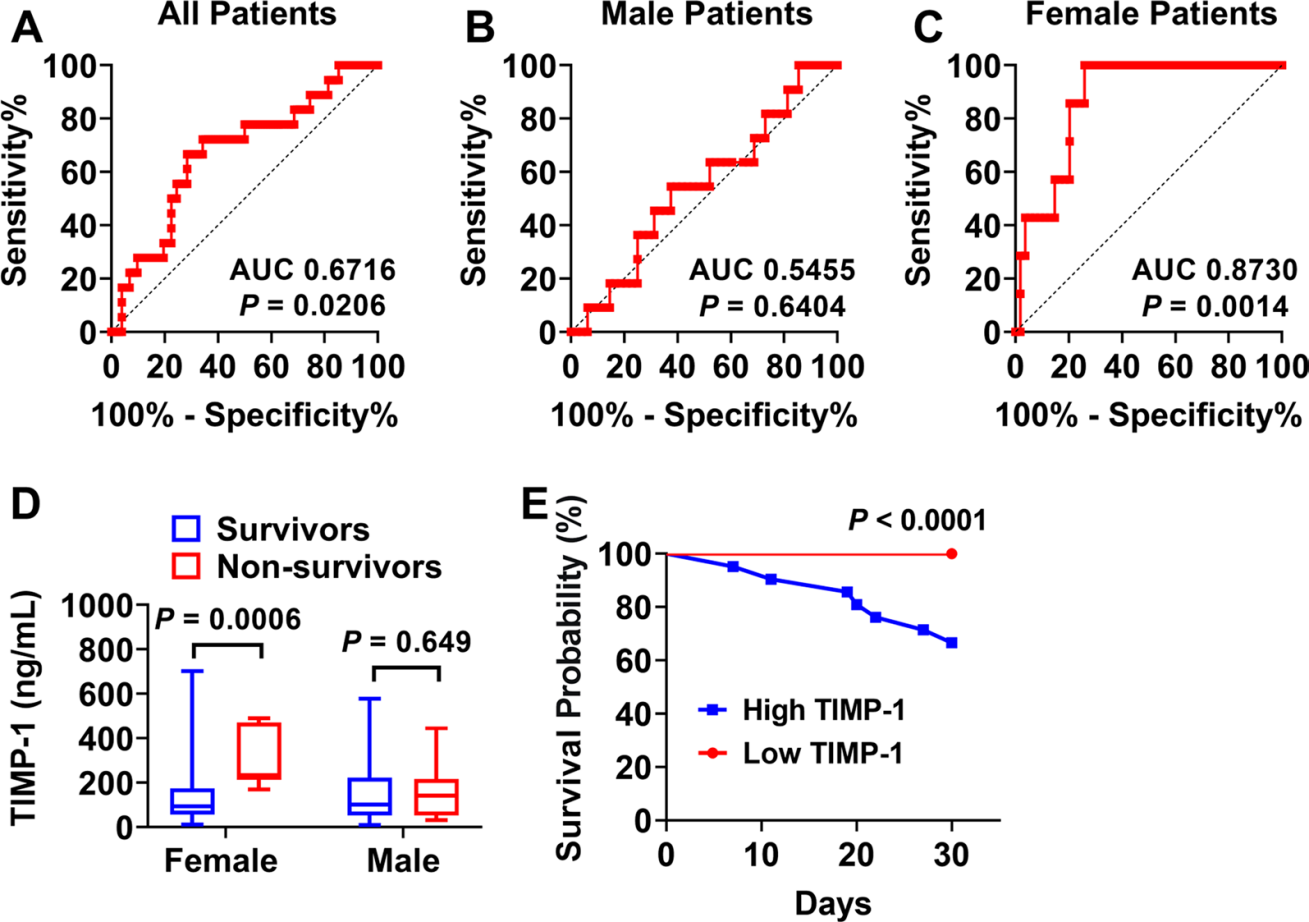
Plasma TIMP-1 levels and 30-day mortality. **A** ROC curve for TIMP-1 concentration and 30-day mortality of all patients (survivors = 82; non-survivors = 18). **B** ROC curve for TIMP-1 concentration and 30-day mortality of male patients (survivors = 43; non-survivors = 11). **C** ROC curve for TIMP-1 concentration and 30-day mortality of all female patients (survivors = 39; non-survivors = 7). **D** Sex divided TIMP-1 level comparison in survivors (females *n* = 39; males *n* = 43), and non-survivors at 30 days (females *n* = 7; males *n* = 11). **E** Kaplan–Meier survival curves for ARDS female patients censored at 30 days

Furthermore, we assessed the TIMP-1 levels in relation to 30-day mortality. Among all ARDS patients, TIMP-1 demonstrated a poor AUROC for the prediction of 30-day mortality (AUC 0.67) (Fig. 2A). Similarly, TIMP-1 demonstrated a poor AUROC among males only (AUC 0.54) (Fig. 2B). Unexpectedly, an excellent AUC with an optimal cut-off value of 159.7 ng/mL producing a 100% sensitivity and 74% specificity was seen after stratifying females (AUC: 0.87, 95% confidence interval [CI] 0.78 to 0.97; *P* = 0.0014) (Fig. 2C). Furthermore, female non-survivors had significantly higher plasma TIMP-1 levels when compared to female survivors on day 30 (female survivors 93.33 ng/mL vs. female non-survivors 233.7 ng/mL, *P* = 0.0006), but not in male subgroup (male survivors 101.5 ng/mL vs. male non-survivors 140.3 ng/mL, *P* = 0.649) (Fig. 2D). When female subjects were grouped as high (≥ 159.7 ng/mL) or low (< 159.7 ng/mL), high TIMP-1 patients exhibited increased 30-day mortality (33% vs 0%, *P* < 0.0001) and significantly increased odds of mortality using time-to-event analysis censored at 30-day follow-up in comparison to the low TIMP-1 group (Fig. 2E).

**Fig. 3:**
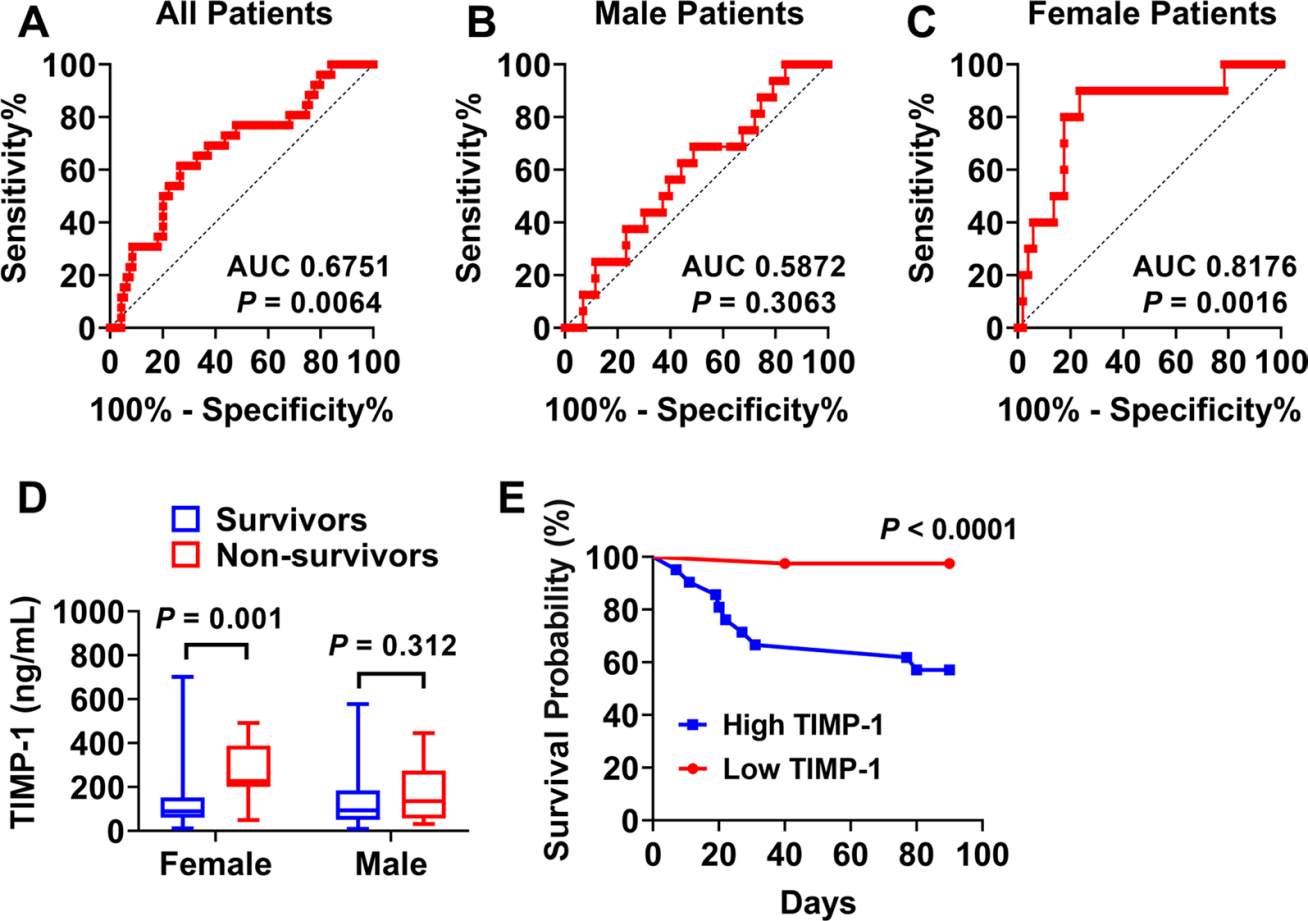
Plasma TIMP-1 levels and 90-day mortality. **A** ROC curve for TIMP-1 concentration and 90-day mortality of all ARDS patients (survivors = 74; non-survivors = 26). **B** ROC curve for TIMP-1 concentration and 90-day mortality of male patients (survivors = 38; non-survivors = 16). **C** ROC curve for TIMP-1 concentration and 90-day mortality of female patients (survivors = 36; non-survivors = 10). **D** Sex divided TIMP-1 level comparison in survivors (females *n* = 36; males *n* = 38), and non-survivors at 90 days (females *n* = 10; males *n* = 16). **E** Kaplan–Meier survival curves for ARDS female patients censored at 90 days

In relation to 90-day mortality, TIMP-1 also demonstrated poor AUROC for the prediction of 90-day mortality among all ARDS patients (AUC 0.67) (Fig. 3A) and males (AUC 0.58) (Fig. 3B). Among the female patients, AUROC analysis showed that TIMP-1 has an AUC of 0.82 (95% confidence interval [CI] 0.67 to 0.97; *P* = 0.0016) with a similar cut-off value producing a 90% sensitivity and 76% specificity (Fig. 3C). Consistently, we found female non-survivors had significantly higher plasma TIMP-1 levels when compared to female survivors on day 90 (female survivors 89.62 ng/mL vs. female non-survivors 226.4 ng/mL, *P* = 0.001), but not in male subgroup (male survivors 92.36 ng/mL vs. male non-survivors 135.1 ng/mL, *P* = 0.312) (Fig. 3D). Similarly, high TIMP-1 was associated with increased risk of 90-day mortality (43% vs 2.5%, *P* < 0.0001) when female subjects were grouped as high (≥ 159.7 ng/mL) or low (< 159.7 ng/mL) (Fig. 3E).

### Plasma TIMP-1 level and association with other relevant clinical outcomes

We grouped the patients into low vs high TIMP-1 based on the above cut-off level and then stratified all patients, males, or females to assess the other relevant clinical outcomes. Among all patients, the high TIMP-1 group had worse ICU-free days (15.17 vs 11.45, *P* = 0.047) but no significant difference in other outcomes (Table 2). Among males, no significant difference was seen between the groups in clinical outcomes (Table 3). Interestingly, both VFDs and ICU-free days outcomes were significantly different between the groups among females (18.48 vs 12.43, *P* = 0.043) and (17.32 vs 11.43, *P* = 0.039), respectively (Table 4).

**Table 2:** Demographic and summary characteristics of ALTA sub-groups based on TIMP-1 levels (all patients)

Characteristic	Low TIMP-1 (≤ 159.7 ng/mL) (*n* = 60)	High TIMP-1 (> 159.7 ng/mL) (*n* = 40)	*P* value
Age	50.87 ± 2.07	50.60 ± 2.36	0.934
APACHE III score	87.12 ± 3.37	99.75 ± 5.02	0.032
PaO_2_/FIO_2_	138.53 ± 7.82	147.13 ± 9.38	0.485
Patient outcomes			
Ventilator-free days	16.27 ± 1.24	12.45 ± 1.70	0.066
ICU-free days	15.17 ± 1.09	11.45 ± 1.55	0.047
Organ failure-free days	14.87 ± 1.35	13.60 ± 1.71	0.560

Means and SEMs were reported for continuous variables

*APACHE III score* Acute Physiology, Age and Chronic Health Evaluation; *PaO*_*2*_ partial pressure of oxygen, *FIO*_*2*_ fraction of inspired oxygen

**Table 3:** Demographic and summary characteristics of ALTA sub-groups (male patients)

Characteristic	Low TIMP-1 (≤ 159.7 ng/mL) (*n* = 35)	High TIMP-1 (> 159.7 ng/mL) (*n* = 19)	*P* value
Age	49.00 ± 2.86	45.42 ± 3.56	0.449
APACHE III score	87.62 ± 4.36	103.74 ± 6.80	0.042
PaO_2_/FIO_2_	143.60 ± 11.48	166.90 ± 12.79	0.207
Patient outcomes			
Ventilator-free days	14.69 ± 1.71	12.47 ± 2.43	0.453
ICU-free days	13.63 ± 1.39	11.47 ± 2.20	0.389
Organ failure-free days	14.49 ± 1.69	14.79 ± 2.43	0.917

Means and SEMs were reported for continuous variables

*APACHE III score* Acute Physiology, Age and Chronic Health Evaluation; *PaO*_*2*_ partial pressure of oxygen, *FIO*_*2*_ fraction of inspired oxygen

**Table 4:** Demographic and summary characteristics of ALTA sub-groups (female patients)

Characteristic	Low TIMP-1 (≤ 159.7 ng/mL) (*n* = 25)	High TIMP-1 (> 159.7 ng/mL) (*n* = 21)	*P* value
Age	53.48 ± 2.93	55.29 ± 2.86	0.665
APACHE III score	86.42 ± 5.42	96.14 ± 7.40	0.287
PaO_2_/FIO_2_	131.45 ± 9.79	129.24 ± 12.65	0.889
Patient outcomes			
Ventilator-free days	18.48 ± 1.73	12.43 ± 2.41	0.043
ICU-free days	17.32 ± 1.70	11.43 ± 2.25	0.039
Organ failure-free days	15.40 ± 2.26	12.52 ± 2.44	0.392

Means and SEMs were reported for continuous variables

*APACHE III score* Acute Physiology, Age and Chronic Health Evaluation; *PaO*_*2*_ partial pressure of oxygen, *FIO*_*2*_ fraction of inspired oxygen

## Discussion

In this observational study, we reported that TIMP-1 as a sex-based biomarker is associated with mortality and other relevant clinical outcomes, VFDs, and ICU-free days. The female sex serves as a significant factor that alters circulating TIMP-1 concentrations and reflects disease progression. Our novel findings support TIMP-1 as a sex-specific predictor for ARDS mortality.

The inflammatory response to lung injury propagates from multiple factors with pathologic synergy leading to the varying severity and phenotypes of ARDS [9]. Previous studies have linked sex as a significant contributor to immunologic response due to sex hormones mediating properties [14–16]. In goat oviductal epithelial cells, estradiol can induce TIMP-1 expression [23]. A similar observation was reported in human aortic endothelial cells under inflammatory conditions but not normal conditions [24]. Thus, there is a possibility that *TIMP-1* may be a gene that escapes X-chromosome inactivation during inflammatory events leading to severe ARDS. This could explain our observation that plasma TIMP-1 is higher in female non-survivors (Figs. 2D and 3D).

Circulating TIMP-1 has not been broadly evaluated as a non-invasive blood marker for lung injury. In clinical cohorts, TIMP-1 systemic level was significantly higher in ARDS subjects than in other sub-groups of ventilated patients with respiratory failure, and it was independently associated with 90-day mortality and worse hypoxemia [28]. Likewise, higher plasma of TIMP-1 concentration was significantly associated with ARDS and 30-day mortality risk in critically ill patients admitted to the ICU [29]. These findings demonstrate the prognostic potential of circulating TIMP-1 in ARDS and encourage further biomarker studies to be conducted. In preclinical studies, both influenza infection and LPS treatment can significantly induce the expression of TIMP-1 in the murine lungs, suggesting that TIMP-1 participates in the pathogenesis of ALI [30–32]. Functionally, *Timp-1* deficiency in mice amplifies bleomycin or LPS-induced acute lung injury [33], suggesting TIMP-1 has a protective role in the lungs. Consistently, *Timp-1*-deficient mice experienced significantly less weight loss compared to wild-type mice after influenza infection [30]. Besides, *Timp-1*-deficient mice demonstrated less immune cell infiltration and airway inflammation [30].

In ALI/ARDS, several causes may contribute to the altered TIMP-1 levels in circulation. For instance, TIMPs and MMPs are thought to contribute to leukocyte influx and vascular permeability at sites of lung injury leading to alteration levels in circulation [30, 34]. Moreover, the expression of TIMP-1 could be altered in circulation due to the remodeling or destruction of the ECM in the lung either by TIMP-1 and MMPs imbalance or other unrecognized mechanisms. The inflammatory role of TIMP-1 is another potential reason for its alteration in biofluids. For example, it has been recently reported that TIMP-1 triggered neutrophil extracellular traps (NETs) formation in patients with pancreatic cancer and there was a significant correlation between TIMP-1 and DNA-bound myeloperoxidase, a NET marker in the plasma [35]. Indeed, this pathogenic mechanism is well recognized in the literature for the development of ALI [36]. These are possible explanations to illustrate how circulating TIMP-1 could change with the progression of lung injury.

TIMP-1 is known to regulate ECM proteolysis via inhibition of MMP-dependent matrix proteolysis but is also assumed to play divergent roles in ECM turnover which is described by matrix accumulation and breakdown [37]. It is also suggested that elevated levels of TIMP-1 than MMPs lead to fibrosis whereas lower TIMP-1 level is associated with proteolysis and fluid leakage [38, 39]. During the early stage of lung injury, sterile or infectious stimuli induce lung inflammation via epithelial/endothelial damage, destruction of the air–blood barrier which ultimately causes the vascular leak, and accumulation of fluid in the alveoli. These could be explained by more matrix degradation as a result of more production of MMPs and low concentrations of TIMP-1 in lung tissue. Later, a provisional matrix can be formed due to profibrotic cytokines and fibroblast proliferation. Then, exaggerated matrix accumulation and lack of matrix degradation can induce progressive lung remodeling or fibrosis, which might be related to elevated expression of TIMP-1 in the lung [40]. Thus, maintenance of the TIMPs–MMPs balance is essential to normal lung function, and exploring the potential of TIMPs–MMPs as therapeutic targets for lung diseases is urgently needed [41]. Currently, TIMP-1 is known to strongly inhibit many MMPs except for some of the membrane-type MMPs like MMP-14, -15, -16, -19, and -24 [37].

Circulating MMPs have not been extensively studied in the aspect of lung injury and only a few studies have assessed MMPs in relation to ARDS severity or mortality. In a retrospective observational study, plasma MMP-9 activity was negatively correlated with PaO_2_/FiO_2_ ratio among patients who developed ARDS compared to other sub-groups of ARF indicating the possibility of circulating MMPs to reflect lung injury [42]. In patients with severe COVID-19, both MMP-2 and MMP-9 levels in circulation were altered and associated with the risk of in-hospital death [43]. Circulating MMP-9 was further measured in COVID-19 patients in addition to the MMP-3 to see if they could predict the severity of the disease as assessed by the World Health Organization (WHO) severity stage. MMP-3 was significantly increased and associated with the progression of the WHO stage while circulating MMP-9 raised but did not show an association with disease severity suggesting heterogeneity of MMPs in response to disease pathogenesis [44]. In patients with ventilator-associated pneumonia (VAP), circulating MMP-9 was significantly elevated in VAP patients and positively correlated with WBCs and neutrophils counts [45]. These reports and the current study encourage further examination of TIMP–MMPs as potential non-invasive blood markers or therapeutic targets for lung injury.

ARDS severity can be assessed based on physical exam, chest X-ray, and most importantly the degree of hypoxemia as evaluated by the PaO_2_/FIO_2_ ratio [46]. Circulating biomarkers such as inflammation proteins, lung endothelium as well as lung epithelium-specific proteins have been examined so far for lung injury [47, 48], but none of them can provide a robust diagnostic or prognostic assessment for ARDS [49]. So far, Krebs von den Lungen-6 (KL-6), primarily secreted by type II alveolar epithelial cells, has been shown an excellent discrimination AUC value among ARDS patients in one cohort [50]. A meta-analysis assessed several blood proteins like surfactant protein-A (SP-A), Club Cell Protein 16 (bronchial epithelial cells), sRAGE (alveolar injury marker), and IL-10 (inflammation marker) and reported that no significant association between these proteins and mortality in ARDS patients [51]. TIMP-1 in the current study showed an excellent discrimination AUC value after stratifying female patients. Due to heterogeneity in ARDS, this study provides novel insights into an aspect of sex-specific morbidity and mortality, suggesting sex needs to be considered in biomarker studies.

Traumatic injury is a common etiology of ARDS among both male and female sex with no significant difference between the groups recognized in the literature [4, 52]. In our current study, the percentage of trauma-induced ARDS was significantly higher in males than females. Likewise, Kasotakis et al. have reported higher percentages of males than females in trauma patients with ARDS [11]. Similarly, the number of males was significantly higher than females in a study aimed to assess the ARDS development in trauma patients [9]. These results suggest that the number of male trauma patients could change the overall percentages of ARDS development and mortality. It also indicates that the male sex might be a prominent confounder for trauma-induced lung injury and mortality.

Our study has limitations, namely the retrospective observational design prevents determining the cause and effect of TIMP-1 and outcomes in female ARDS. Moreover, TIMP-1’s biological significance appears restricted to females and the number of patients in the female subgroup is relatively small. This study neither evaluates the biological roles of TIMP-1 in ARDS nor correlates plasma TIMP-1 to injury levels in the lung. Thus, it is unknown whether TIMP-1 indicates more severe ARDS or contributes to its development. Nevertheless, our study provides new insights into sex-based biomarkers in ARDS.

## Perspectives and significance

In this study, we examined circulating TIMP-1 in lung injury from a prospective randomized controlled trial. TIMP-1 appeared to be a promising biomarker for mortality in females with ARDS and was also associated with other relevant clinical outcomes, including VFDs and ICU-free days. Our data suggest that TIMP-1 as a sex-specific protein might not only be a sex-based biomarker in the setting of lung injury, but also could be a targeted therapy in female ARDS patients. Further investigations are needed to address the regulation of TIMP-1 and its sex-specific role in the pathogenesis of ARDS.

## Data Availability

The datasets used and/or analyzed during the present study are available from the corresponding author upon reasonable request.
